# Combination Effect of Notch1 and PI3K/AKT/mTOR Signaling Pathways Inhibitors on T-ALL Cell Lines

**Published:** 2020-04-01

**Authors:** Halimeh Khoshamooz, Saeid Kaviani, Amir Atashi, Seyed Hossein Mirpour Hassankiadeh

**Affiliations:** 1 Department of Hematology and Blood Banking, Faculty of Medicine, Tarbiat Modares University, Tehran, Iran; 2 Department of Hematology, Faculty of Medicine, Tarbiat Modares University, Tehran, Iran; 3 Department of Basic Sciences, School of Medicine, Shahroud University of Medical Sciences, Shahroud, Iran; 4 Department of Internal Medicine, School of Medicine, Guilan University of Medical Sciences, Rasht, Iran

**Keywords:** Synergistic interaction, AZD5363, Compound E, Acute T-lymphoblastic leukemia

## Abstract

**Background:** Acute T lymphoblastic Leukemia (T-ALL) is a highly aggressive hematologic malignancy. Chemotherapy resistance is one of the most important challenges in T-ALL treatment. Alterations in cellular signaling pathways such as Notch1 and PI3K/AKT/mTOR play a role in cell proliferation, survival, and resistance to chemotherapy. Combination of Notch1 and PI3K/AKT/mTOR inhibitors is an interesting and rational strategy in treatment of T-ALL. Interaction of AZD5363 as an inhibitor of PI3k/AKT/mTOR and Compound E as an inhibitor of Notch1 signaling pathway was investigated in a T-ALL pre-clinical model.

**Materials and Methods:** T-ALL cell lines included Jurkat, Molt-4, and HPB- ALL cells were treated with AZD5363 and Compound E alone and in combination. Cell viability was determined by MTT assay. Flow cytometry was used to measure apoptosis**.** Interaction between AZD5363 and Compound E was assessed by Chou-Talalay method.

**Results:** Combination treatment with AZD5363 and Compound E decreased cell viability with synergistic effect in all cell lines at 72 hours. Drug combination increased apoptosis even in Jurkat and HPB-ALL cells resistant to Compound E and AZD5363, respectively.

**Conclusion:** Combination of AZD5363 with Compound E in T-ALL cell lines exhibited a synergistic effect. Cytotoxicity of drug combination increased in all T-ALL cell lines compared to each as a single drug. Simultaneous inhibition of Notch1 and PI3K/AKT signaling pathways as a possible treatment of T-ALL, provides a basis for future investigations.

## Introduction

 Acute T lymphoblastic (T-ALL) is a highly aggressive hematologic malignancy in which at least 25% of bone marrow mononuclear cells are occupied by T lymphoblasts^1^. T-ALL account about 10-15% and 25% of pediatric and adult acute lymphoblastic leukemia, respectively^2^. Clinical features suggest that many white blood cells are due to the proliferation of T cell lymphoblasts infiltrate to mediastinum and central nervous system^3^. Intensive chemotherapy can induce remission in 50% and 75% of adults and Children, respectively. Chemotherapy resistance is one of the most important challenges in T-ALL treatment^   4 ^.

Discovery of new drugs with selective properties for the tumor and lower toxicities for normal cells, as well as the use of drug combinations that target different pathways in cancer cells can be effective in patient outcome and improving therapeutic effects^   5 ^.

Alterations in cellular signaling pathways play a major role in etiology, maintenance and progression of T-ALL. One of the important signaling pathways in T-ALL is Notch1 signaling^6^. About 50-60% of T-ALL cell lines and primary T-ALL cells harbor activating mutations in *Notch1* gene^7^. Notch1 is a membrane-receptor protein with oncogenic properties, including cell proliferation, survival, and resistance to chemotherapy. Inhibition of Notch1 activity by small molecules gamma-secretase inhibitors (GSIs) is one of the strategies for T-ALL target therapy. Since these compounds are not effective alone, they can be combined with agents that inhibit the other targets^   8 ^. 

The PI3K/AKT/mTOR signaling pathway is constitutively active in approximately 50-75% of T-ALL patients, which has a poor prognosis. This signal transduction cascade plays a role in cell growth, survival and drug resistance of T-ALL. Therefore, inhibition of PI3K/AKT/mTOR signaling can be effective in T-ALL targeted therapy^9^. Clinical trials have shown that a strong response to this pathway inhibitors is rare. It is necessary to combine them with other drugs to improve therapeutic efficacy by inducing synergy and reducing drug toxicity^   10 ^.

Combination of Notch1 and PI3K/AKT/mTOR inhibitors is an interesting and rational strategy in treatment of T-ALL^11^. Activation of both signaling pathways in T-ALL patients and functional interactions between them provides valuable reasons for using them as a combination strategy in T-ALL therapy. It is important to note that some T-ALL cells with active Notch1 are resistant to Notch1 inhibitors via the PI3K/AKT/mTOR signaling pathway. Therefore, adding PI3K/AKT/mTOR inhibitors to Notch1 inhibitors may prevent drug resistance to Notch1 inhibitors ^12^. Another important point is that both of them have a role in proliferation and survival of T-ALL cells. There is cross-talk between the pathways. For example, Notch1 signaling interaction with PI3K/AKT/mTOR pathway promote cell growth induced by active Notch1^   13 ^.

In order to study the inhibition of Notch1 and PI3K/ AKT/mTOR signaling pathways simultaneously in T-ALL cell lines, a gamma secretase inhibitor called Compound E and a strong selective AKT inhibitor, AZD5363 were used. Drugs effect on cell viability and induction of apoptosis was assessed by MTT assay and flow cytometry, respectively.

## MATERIALS AND METHODS

**Cell lines and cell cultures**

Human T-ALL cell lines were purchased from the Pasteur Institute of Iran:

     •    Molt-4(NCBI code: C149, ATCC Number:CRL-1582) 

     •    Jurkat E6.1(NCBI code: C121, ATCC Number:TIB-152) 

     •    HPB-ALL(NCBI code: C213, DSM Number:ACC-483)

According to the Sanger Cosmic database, molt-4 cell line has a heterozygous mutation in* NOTCH1* gene and the homozygous mutation in *PTEN* (*PTEN*-null). HPB-ALL cell has both mutations in *FBXW7* and *NOTCH1*, but has a *PTEN *wild type (*PTEN* +). JURKAT cell line is *PTEN* null. All cells were cultured in RPMI-1640 medium with 10% FBS and 2 mM L-glutamine and 100 U/ml penicillin and 100 mg/mL streptomycin, and incubated in 5 % CO2 incubator at 37 ° C.

**Drugs**

- Compound E :((S,S)- 2-[2-(3,5-Difluorophenyl)-acetylamino]-N-(1-methyl- 2-oxo-5-phenyl-2,3-dihydro-1H-benzo[e][1,4]diazepin-3-yl)-propionamide (Santa-cruez,Texas ,USA )^   14 ^
- AZD5363:[(S)-4-amino-N-[1-(4-chlorophenyl)-3-hydroxypropyl]-1(7H-pyrrolo[2,3-d] pyrimidin-4-yl) piperidine-4-carboxamide(cayman, Michigan, USA)^     15 ^

Drugs purchased and dissolved in dimethyl sulfoxide solution (DMSO) and aliquots were stored at-20C^°^.

**Viability (MTT) assay**

In a 96-well microplate, 1× 10^4^ of Jurkat cells, 4 × 10^4^ of molt-4 cells, 1 × 10^4^ of HPB-ALL cells (in logarithmic phase) were added to each well. Cells treated with different concentrations of AZD5363 (0.5, 1, 10, 20 μM) and Compound E (1.10, 15.20μM) .The microplate was incubated for 24, 48, and 72 hours. Vincristine was used as a positive control. DMSO was used as control for untreated cells. After incubation, the microplates were centrifuged for 5 Minutes with 1200 RPM. The supernatant fluid was discarded. Then, 100 μL of the culture medium was added and mixed. 10 μL of MTT solution (Carl Roth, GmbH, Germany) with 1 mg / mL concentration in the PBS buffer was also added. The plates were incubated for 3 to 4 hours in a 5% CO2 incubator at 37 ° C. Then, they were centrifuged for 5 minutes. The plate was covered with foil and placed at room temperature for 15 minutes.

In a dark environment, 50μL of DMSO solution was added to each well, and the optical density was read using an ELISA reader (BioTek, USA) against control (DMSO) at wavelength of 570 nm versus a 630nm as reference. Each optical absorbance at 570 nm was subtracted from absorption at 630 nm. The experiment was performed on three replicates and repeated three times.

**Combination index assay**

Experimental design of the combination of two drugs for synergy or antagonism effects requires to determine the power or potency and shape of the dose-effect curve for each drug. The parameters of m_1_ and Dm_1_, m_2_, and Dm_2_ calculated for each drug individually, as well as Dm_1, 2_and m_1, 2_ and the CI or Combination Index for drug combination. Dm is median of the drug effect such as IC_50_, in which the drug concentration can inhibit 50% of cell growth. m is a coefficient that actually indicates the shape of the dose-effect curve^   16 ^.

In this study, a fixed constant ratio designing was used to assess drug combination. Two signaling inhibitors were added simultaneously to the cells. First, a mixture of the inhibitors in a constant ratio was prepared and serial dilutions was used. The combination incubation was similar to the single incubation of each inhibitor alone^   17 ^.In order to investigate the effect of the drugs, Combination index (CI) was calculated by using the compusyn software. CI <1 shows synergistic effects of the drugs. CI = 1 represents additive effect and CI> 1 is evidence of the antagonistic effect of the two drugs^   18 ^.

**Apoptosis by flow cytometry**

At the onset of apoptosis, phosphatidylserine translocates to external portion of the plasma membrane. Phosphatidylserine binds to the annexin V-FITC conjugate in presence of calcium.

In apoptosis assay, binding of annexin V-FITC to phosphatidylserine in membrane of cells, and binding of propidium iodide to the cellular DNA were measured.

Annexin V-FITC apoptosis detection kit (Sigma) was used. 500 μl of the apoptotic cell suspension to a plastic 12 × 75 mm test tube was added. 500 μl of the non-induced cell suspension was added to a second test tube. Then, 5 μl of Annex in V- FITC Conjugate and 10 μl of Propidium Iodide Solution were added to each cell suspension. The tubes were incubated at room temperature for 10 minutes and protected from light.

The fluorescence of the cells immediately was determined with flow cytometer (FACS Calibur, BD San Jose, CA, USA). Early apoptotic cells stained with the Annexin V-FITC Conjugate alone. Live cells showed no staining by either the Propidium Iodide Solution or Annexin V-FITC Conjugate. Necrotic cells stained by both the Propidium Iodide Solution and Annexin V-FITC Conjugate. Data were analyzed by using FlowJo software.

**Statistical analysis**

Drug effects both alone and in combination was evaluated using CompuSyn Software (version1.0). Statistical differences between control and drug treated cells were determined by one-way analysis of variance (ANOVA). Values less than 0.05 were considered significant. Data were analyzed by using GraphPad Prism (Version 8.0.1)

## Results

**Effect of Notch1 pathway inhibitor on cell viability and apoptosis**

**Figure 1:**
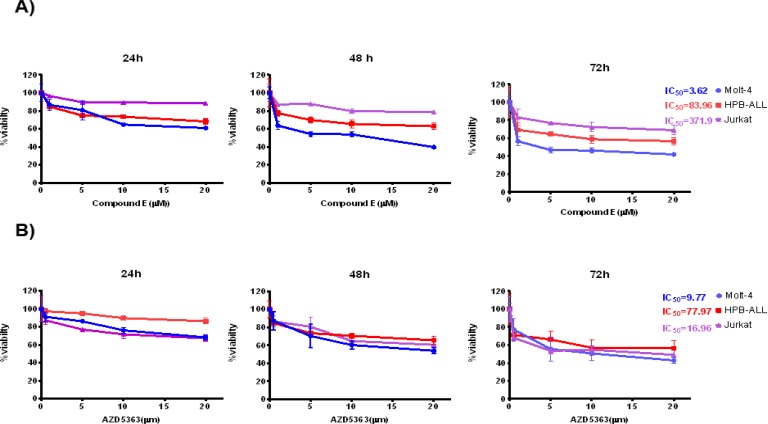
**A)** Molt-4, HPB-ALL, Jurkat were treated with different concentrations of Compound E (from 1 to 20 μM) for 24, 48, and 72 hours. B) HPB-ALL, Molt-4 and Jurkat cells were treated with different concentrations of AZD5363 (from 0.5 to 20 μM) for 24, 48, and 72 hours. . IC_50_ was obtained by MTT assay. Data present in mean. The values represents three independent experiments (Nonlinear regression).

We used Compound E, a potent gamma secretase inhibitor, to block Notch1-mediated signal transduction in T-ALL cell lines. The direct effect of Compound Eon T-ALL proliferation was assessed using three T-ALL cell lines (Molt-4, HPB-ALL and Jurkat). Cell viability was measured by MTT after treatment for 24h, 48h and 72h with 1nM, 5nM, 10nM, 20nM, Compound E, respectively. Inhibitory effect was observed in a dose-dependent and time-dependent manner .The greatest inhibitory effect of Compound E on cell viability was observed 72 hours after treatment. Compound E inhibited potently in vitro cell viability in Molt-4 cells (IC_50 _= 3.62±0.94nM). HPB-ALL cells showed partial inhibition on cell viability even after 72 hours, but did not reach IC_50_. Jurkat was resistant to compound E (Figure 1A).

**Figure 2:**
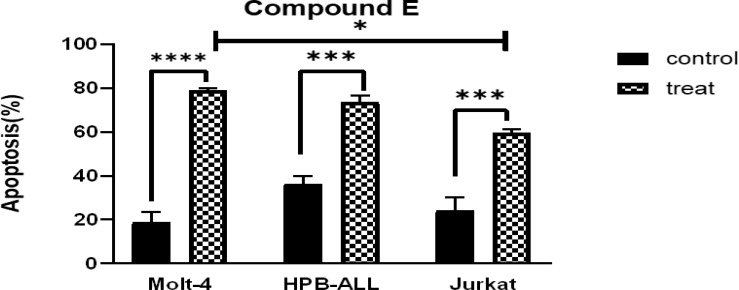
Comparison of % annexin Vs-positive cells in T-ALL cell lines after 72 hours exposure to 5 µM Copmound E for Molt-4,25µM HPB-ALL,80 µM for Jurkat .***p<0.0004,****P<0.0001.(one-way ANOVA and Tukey multiple comparison test).

Apoptosis was induced in all T-ALL cell lines treated with Compound E concentrations for 72 hours (Figure 6). There was a significant difference between treated cells and control in Molt-4 cells (P <0.0001) (Figure. 2).

**Effect of PI3k/Akt/mTOR pathway inhibitor on cell viability and apoptosis**

The cells were treated at concentrations of 0.5, 5, 10, 20 μM of AZD5363 and incubated for 24, 48, 72 hours. Inhibition of survival was observed in a dose-dependent and time-dependent manner. The best inhibitory response was observed 72 hours after treatment. AZD5363 suppressed cell viability in Jurkat and Molt-4 cell lines. AZD5363 potently inhibited Molt-4 cells survival. HPB-ALL cells were resistant to AZD5363. The IC_50 _range, the concentration of AZD5363 which inhibited the survival of 50% of the cells, was (9. 8-77.8 nM) after 72 hours (Figure 1B).

Apoptosis was induced by AZD5363 on all three T-ALL cell lines (Figure 6). However, in Jurkat and Molt-4 cells, there was more significant difference between treated cells with their control group than HPB-ALL (P<0.0001 vs p=0.006) (Figure. 4).

**Figure3:**
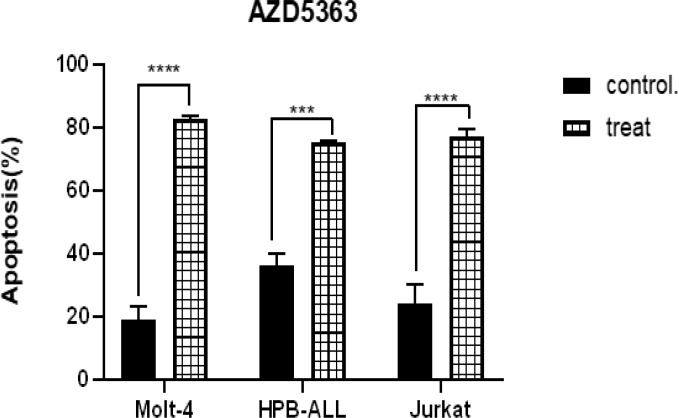
Comparison of % Annexin V-positive cells in T-ALL cell lines after 72 hours exposure to 15 µM AZD5363 for Molt-4,20µM for HPB-ALL,10µM for Jurkat cells.***p=0.0006,****p<0.0001. (One-way ANOVA and Tukey multiple comparison test).

**Combination effect of Notch1 and PI3K/AKT /mTOR signaling pathways inhibitors on cell viability and apoptosis**

Three concentrations with a constant ratio proportional to IC_50_value of the single drug were used for each cell line. In Molt-4, HPB-ALL and Jurkat cells, total dose of 5- 10-20μM, 11.25-22.5-45 μM and 22-45-90μM were applied, respectively. The constant ratio of AZD5363 to Compound E was 3:1 in Molt-4, 4:5 in HPB-ALL and 1:8 in Jurkat cells, at first, the highest concentrations were prepared for each cell line and then serial dilution. MTT assay was performed for each combination in three replicates with control and blank at 72 hours.

**Figure 4:**
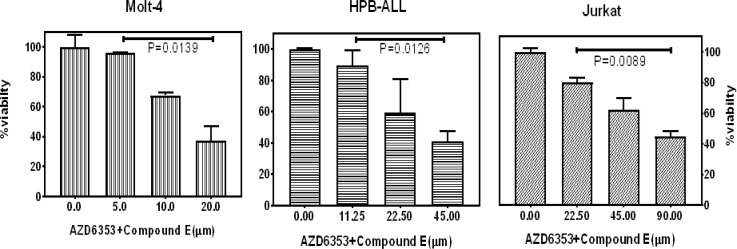
Molt-4, HPB-ALL and Jurkat cells were treated with three concentrations of AZD5363 and Compound E in a constant ratio proportional to IC_50 _ of each drug and incubated for 72 hours.Drug combination effect was assessed by MTT assay .The combination reduced cell viability in T-ALL cells compared to control in a dose-dependent manner. Data are expressed as mean±SEM .The values represents three independent experiments. (One sample t-test)

**Figure 5:**
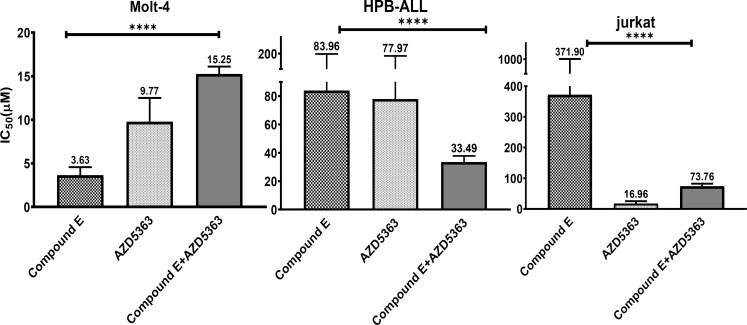
IC50 of drug combination vs AZD5363 or Compound E alone after 72 hours incubation in the three cell lines was determined by MTT assay. Data expressed in mean. All experiments were repeated for three times independently.****p<0.0001 (One-way ANOVA).

Cell viability decreased significantly in three T-ALL cell lines in a dose-dependent manner (Figure 4). Minimum and maximum IC_50_ value of drug combination belonged to Molt-4 and Jurkat cells, respectively. IC_50_ value of drug combination significantly decreased compared to IC_50 _of each drug alone in all cell lines (p<00001) (Figure 5).

The combination of AZD5363 and Compound E showed synergistic effects in T-ALL cell lines. All cell lines in fraction affected or f _a_<0.5 had a combined index less than 1. This synergistic effect was observed in all cell lines even in the Jurkat cells which exhibited resistance to Compound E. Moreover, HPB-ALL cells that were resistant to AZD5363 and partially resistant to Compound E showed maximum synergy (Table 1).

**Table 1:** The combination indices (CI) of drug combination

	**Molt-4**	**HPB-ALL**	**Jurkat**
Dose	15μg AZD5363+ 5μg Compound E	20μg AZD5363+ 25μg Compound E	10μg AZD5363+ 80μg Compound E
CI*	0.535±0.05	0.074±0.008	0.320±0.04

* Combination index (CI) was obtained from the Compusyn software. CI<1 indicated synergism. Data are expressed as mean ± SEM.

**Figure 6:**
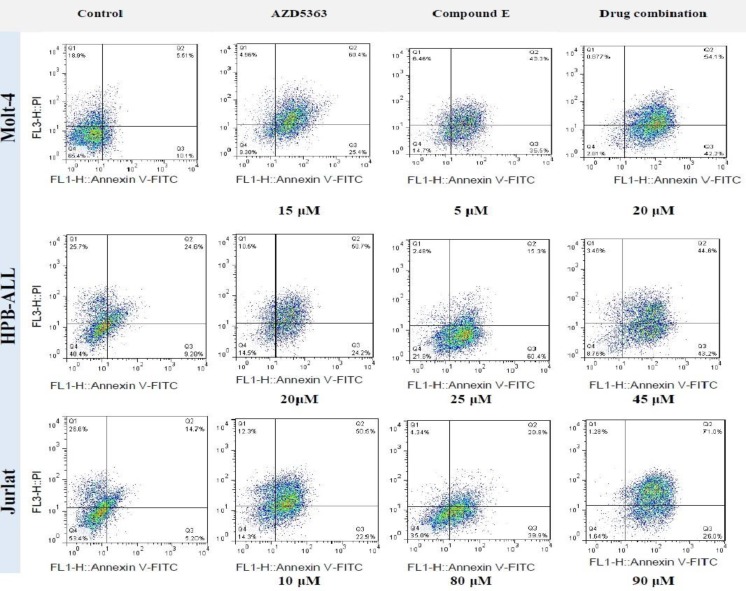
Apoptosis induction by AZD5363 and Compound E as a single and AZD5363 + Compound E combination .Cells were treated with different concentrations of drugs or equal volume of DMSO (control) for 72 hours. Apoptotic cells were stained with AnnexinV / PI staining. Data obtained from three Independent experiments

**Figure 7:**
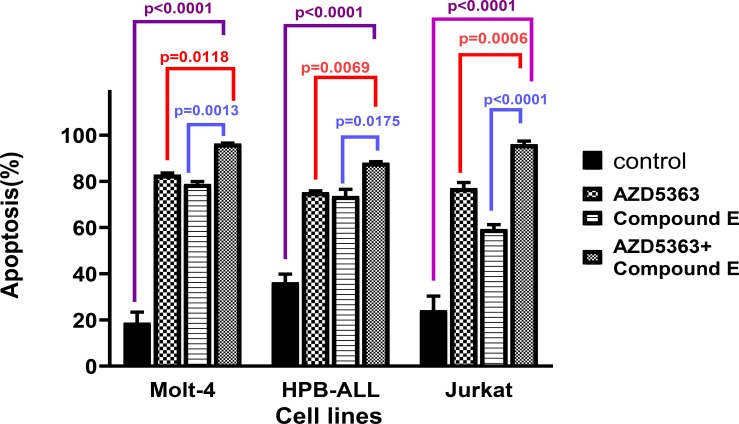
Percentage of apoptotic cells in three cell lines treated with AZD5363 and Compound E both alone and in combination for 72 hours.*P <0.03 ,**P<0.020,***P<0.001,****P<0.0001. (One-way ANOVA and Tukey multiple comparison test).

The combination of Compound E and AZD5363 induced apoptosis in the three cell lines. (Figure 6). Annexin V-positive cells (Early and late apoptosis) increased after 72 hours treatment with AZD5363 and Compound E combination in all cell lines compared to control (p <0.0001). GSI resistant Jurkat cells showed higher apoptosis when treated with drug combination compared to AZD5363 or Compound E alone (P<0.006 vs AZD5363, P<0.0001 vs Compound E) (Figure 7).

**Table 2:** Mean of %apoptosis induced by AZD536, Compound E, and drug combination in T-ALL cell lines.

**Drug**	**AZD5363**	**Compound E**	**AZD5363+Compound E**
%apoptosis	78.43±2.293	70.63±5.842	93.52±2.710

Data are expressed as mean ± SEM.

The comparison of different cell lines showed that induction of apoptosis by the combination drugs in the Molt-4 and Jurkat cell lines had a significant difference compared to control. (P <0.0001). Jurkat cells with higher IC_50_ value ​​than the others showed higher apoptosis when treated with AZD5363 and Compound E combination (P <0.0001) (Figure 7). Maximum apoptosis (96.4%) was induced by 20μg drug combination in Molt-4 cells. AZD5363 and Compound E induced apoptosis in more than 70% of the cells alone, but apoptosis was induced more than %90 of the cells treated with the drug combination (Table 2).

## Discussion

 Different oncogenic pathways affect cancer cells, and targeting of a signaling pathway is often not a good response. However, multi-targeting increases anticancer effects^19^. In acute T lymphoblastic leukemia, two important cell signaling pathways involving in proliferation, survival and resistance to chemotherapy are Notch1 and PI3k/AKT/mTOR signaling pathways .The pathways are active in over 50% of patients^20^. Simultaneous targeting of these two pathways can be a suitable therapeutic strategy^21^. In this study, cytotoxic effects of AZD5363, a Pan-AKT inhibitor, and Compound E, a gamma-secretase inhibitor, as a pre-clinical model for T-ALL therapy were evaluated.

It was first established that Compound E alone was able to reduce potently viability of Molt-4 cell line. The viability of HPB-ALL cells was partially inhibited by Compund E. Jurkat cells was resistant. The effect was dose and time dependent. Compound E (γ-secretase inhibitor XXI) is a cell permeable, potent, selective, non-competitive inhibitor of γ-secretase. Lewis et al. addressed that in NOTCH1-dependent T-ALL cell lines, inhibition of gamma secretase associated with decreased formation of intracellular domain of NOTCH1, reduced the activity of NOTCH1 signaling, and diminished cell viability^22^, but Yoon and his team found that some of T-ALL cells with activated Notch1 did not respond to GSI^23^ . Consistent with Yoon, in the present study, all three cell lines had active mutations in *Notch1*, but Jurkat cell line exhibited resistance to Compound E. Keersmaecker et al. demonstrated in Jurkat cells, GSI resistance was associated with loss of PTEN, a tumor suppressor gene and subsequent activation of the AKT pathway. PTEN inhibits PI3K-AKT signaling pathway. Moreover, in GSI-resistant/*PTEN*-null Jurkat cell line, oncogenic addiction to AKT signaling occurs. The cells are depended on high levels of AKT to maintain the malignant phenotype. In other words, secondary addiction is introduced ^24^^, ^^25^. 

HPB-ALL cells with wild-type* PTEN*, were partially resistant to Compound E. In *PTEN*-positive HPB-ALL cells, inhibition of NOTCH1 increased expression of *PTEN*, followed by the PTEN protein level, which inhibited the PI3K-AKT pathway gradually associated with decreasing phosphorylation of Ser473-AKT ^11^.

Interestingly, the most sensitivity to Compound E was observed in Molt-4 cells that were *PTEN*-null. Zuurbier et al. reported that some of the T-ALL cell lines have mutations in *PTEN* responding to gamma-secretase inhibitors ^26^.

AZD5363 is an oral drug and a new generation of protein kinase B or AKT inhibitor that inhibits all its three isoforms. AZD5363 has been tested as an anticancer drug in tumor models such as breast cancer, prostate cancer and leukemia. AKT is a serine / threonine protein kinase, which plays a central role in PI3K /AKT/ mTOR signaling network^27^.

In this study, it was proved AZD5363 reduced viability of Molt-4 and Jurkat cell lines in a dose and time dependent manner. HPB-ALL cells exhibited partial resistance. AZD5363 induced apoptosis in all three cell lines, but Jurkat and Molt-4 cell lines showed more significant difference compared to their control group than HPB-ALL. Jurkat and Molt-4 cells have active PI3K/AKT/mTOR signaling due to mutations in PTEN compared to HPB-ALL cells with no active PI3K/AKT/mTOR .This is consistent with a recent study, which demonstrated that AKT inhibitors induced apoptosis in PTEN null cell lines with AKT activation^28^.

Combination index analysis showed synergistic effects of PI3K/AKT/mTOR and NOTCH-1 inhibitors when used simultaneously in all three T-ALL cell lines. While Jurkat cells were resistant to Compound E, addition of AZD5363 showed a synergistic effect. Jurkat cells due to activation of AKT and oncogenic addiction to PI3K / AKT were GSIs resistant. Therefore, suppression of AKT by its inhibitor was more effective on cell cytotoxicity. Molt-4 cells which harbor *PTEN* loss of function were sensitive to AZD5363 and Compound E alone. Moreover, the drug combination exhibited maximum cytotoxicity.

## CONCLUCION

 The results of this study showed that combination of AZD5363 with Compound E in T-ALL cell lines had synergistic effect. Cytotoxicity of drug combination increased in all T-ALL cell lines compared to each as a single drug. Further studies are needed to examine the mechanism of apoptotic pathways and In vivo studies. In T-ALL cell lines simultaneously targeting of Notch1 and PI3k/AKT/ mTOR signaling pathways can increase the cytotoxicity of the drugs .The combination of AZD5363 and Compound E as a possible treatment of T-ALL provides a basis for future investigations.

## References

[1] Fielding AK, Banerjee L, Marks DI (2012). Recent developments in the management of T-cell precursor acute lymphoblastic leukemia/lymphoma. Curr Hematol Malig Rep.

[2] Bongiovanni D, Saccomani V, Piovan E (2017). Aberrant Signaling Pathways in T-Cell Acute Lymphoblastic Leukemia. Int J Mol Sci.

[3] Marks DI, Rowntree C (2017). Management of adults with T-cell lymphoblastic leukemia. Blood.

[4] Paganin M, Ferrando A (2011). Molecular pathogenesis and targeted therapies for NOTCH1-induced T-cell acute lymphoblastic leukemia. Blood Rev.

[5] Mokhtari RB, Homayouni TS, Baluch N (2017). Combination therapy in combating cancer. Oncotarget.

[6] Hales EC, Taub JW, Matherly LH (2014). New insights into Notch1 regulation of the PI3K–AKT–mTOR1 signaling axis: Targeted therapy of γ-secretase inhibitor resistant T-cell acute lymphoblastic leukemia. Cell Signal.

[7] Girardi T, Vicente C, Cools J (2017). The genetics and molecular biology of T-ALL. Blood.

[8] Tzoneva G, Ferrando AA (2012). Recent advances on NOTCH signaling in T-ALL. Curr Top Microbiol Immunol..

[9] Cani A, Simioni C, Martelli AM (2015). Triple Akt inhibition as a new therapeutic strategy in T-cell acute lymphoblastic leukemia. Oncotarget.

[10] Fransecky L, Mochmann LH, Baldus CD (2015). Outlook on PI3K/AKT/mTOR inhibition in acute leukemia. Mol Cell Ther..

[11] Palomero T, Sulis ML, Cortina M (2007). Mutational loss of PTEN induces resistance to NOTCH1 inhibition in T-cell leukemia. Nat Med.

[12] Mendes RD, Canté-Barrett K, Pieters R (2016). The relevance of PTEN-AKT in relation to NOTCH1-directed treatment strategies in T-cell acute lymphoblastic leukemia. Haematologica.

[13] Roti G, Stegmaier K (2014). New Approaches to Target T-ALL. Front Oncol..

[14] Yoon SO, Zapata MC, Singh A (2014). Gamma secretase inhibitors enhance vincristine-induced apoptosis in T-ALL in a NOTCH-independent manner. Apoptosis.

[15] Zhang Y, Zheng Y, Faheem A (2016). A novel AKT inhibitor , AZD5363 , inhibits phosphorylation of AKT downstream molecules , and activates phosphorylation of mTOR and SMG ‑1 dependent on the liver cancer cell type. Oncol Lett.

[16] Chou TC (2006). Theoretical Basis, Experimental Design, and Computerized Simulation of Synergism and Antagonism in Drug Combination Studies. Pharmacol Rev.

[17] García-Fuente A, Vázquez F, Viéitez JM (2018). CISNE: An accurate description of dose-effect and synergism in combination therapies. Sci Rep.

[18] Goto S, Goto H, Yokosuka T (2016). The combination effects of bendamustine with antimetabolites against childhood acute lymphoblastic leukemia cells. Int J Hematol.

[19] Cosenza M, Civallero M, Fiorcari S (2016). The histone deacetylase inhibitor romidepsin synergizes with lenalidomide and enhances tumor cell death in T-cell lymphoma cell lines. Cancer Biol Ther.

[20] Raetz EA, Teachey DT (2016). T-cell acute lymphoblastic leukemia. Hematology Am Soc Hematol Educ Program.

[21] Bertacchini J, Heidari N, Mediani L (2015). Targeting PI3K/AKT/mTOR network for treatment of leukemia. Cell Mol Life Sci.

[22] Yoon S, Zapata MC, Singh A (2014). Gamma secretase inhibitors enhance vincristine-induced apoptosis in T-ALL in a NOTCH-independent manner. Apoptosis.

[23] Lewis HD, Leveridge M, Strack PR (2007). Apoptosis in T Cell Acute Lymphoblastic Leukemia Cells after Cell Cycle Arrest Induced by Pharmacological Inhibition of Notch Signaling. Chem Biol.

[24] Keersmaecker KD, Lahortiga I, Mentens N (2008). In vitro validation of γ-secretase inhibitors alone or in combination with other anti-cancer drugs for the treatment of T-cell acute lymphoblastic leukemia. Haematologica.

[25] Knoechel B, Roderick JE, Williamson KE (2014). An epigenetic mechanism of resistance to targeted therapy in T cell acute lymphoblastic leukemia. Nat Genet.

[26] Zuurbier L, Petricoin EF, Vuerhard MJ (2012). The significance of PTEN and AKT aberrations in pediatric T-cell acute lymphoblastic leukemia. Haematologica.

[27] Choi AR, Kim JH, Woo YH (2016). Co-treatment of LY294002 or MK-2206 with AZD5363 attenuates AZD5363-induced increase in the level of phosphorylated AKT. Anticancer Res.

[28] Lynch JT, McEwen R, Crafter C (2016). Identification of differential PI3K pathway target dependencies in T-cell acute lymphoblastic leukemia through a large cancer cell panel screen. Oncotarget.

